# US–China science collaboration to solve global grand challenges

**DOI:** 10.1073/pnas.2320207120

**Published:** 2023-12-21

**Authors:** Roger N. Beachy, Karen C. Seto

**Affiliations:** ^a^Department of Biology, Washington University, St. Louis, MO 63130; ^b^Yale School of the Environment, Yale University, St. Louis, MO 63130

Whether it is climate change, food security, or health, humanity faces challenges that cannot be solved by a single country. As the Covid-19 pandemic exemplified, many threats transcend national borders and require scientific collaboration among countries. The urgent need to address global challenges is damaged today by the deteriorating political relationship between China and the United States, with serious effects on science collaboration. Joint scientific work between the two countries is declining: the number of joint research projects that result in co-authored publications and scientific exchanges of scientists and students are trending down. Even long-standing collaborations risk fracturing with today’s deteriorating political climate. And the US–China Science and Technology (S&T) Cooperation Agreement, which fosters collaboration between the two countries and has been routinely approved for nearly 45 years, is hanging on a thread, temporarily extended until February 2024.

Recently, we were among a small group of scientists from the US National Academies of Sciences, Engineering, and Medicine who met with leading scientists from China in Trieste, Italy, to discuss how we might begin to overcome the political impasse and work together on solving grand challenges. Both sides agreed on three things: 1) the need for scientists on both sides to collaborate; 2) developing a framework to ensure long-term scientific interactions; and 3) the urgency of increasing collaborative opportunities for young scientists. Designed to forge new connections and strengthen existing ones, this meeting was supported by the presidents of the US National Academy of Sciences (NAS) and Chinese Academy of Sciences (CAS). In fact, CAS President Hou Jianguo joined us at the opening dinner, stating that, “We believe that no one single country can tackle global challenges of our time on its own, and international cooperation can benefit all partners and accelerate the advancement of science and technology.” And, the week prior to our meeting, NAS President Marcia McNutt sent a message to all NAS members, in which she emphasized that, “we need to identify productive ways to maintain connections between the U.S. and China, especially in mutually beneficial areas.”

Our group is now planning a larger joint US–China meeting in early 2024, tasked with forming a series of working groups in specific areas that seem likely to produce future breakthroughs. Each working group will then organize workshops designed to create collisions between scientists with relevant talent and expertise, with the aim of creating productive collaborations between young future leaders in both nations.

If we are to solve humanity’s most pressing challenges, we must work together. The threats of changes in climate and extreme weather events have devastating impacts on production of food and can change their nutritional value and consequently affect human well-being. Climate change is altering the movement of insects that vector diseases of plants, animals, and humans that result in pandemics with global impacts. Contemporary urbanization has created conditions that require adaptive changes to enhance resilience to changing a climate. Research collaborations between scientists in many nations are necessary to solve these and other global challenges. Collaborations between the United States and China, two science powerhouses, must continue to bring solutions to such challenges to ensure continuing human advancements that can be shared around the world. They will also forge many personal relationships of trust between our two nations that will insure accurate, honest communications and help avoid future misunderstandings. We believe that concrete steps, including a long-term renewal of the S&T agreement, are critical to ensuring a thriving US science community and maintaining collaboration between two science powerhouses in a way that can help solve humanity’s most pressing challenges.

